# Black Tea Lowers Blood Pressure and Wave Reflections in Fasted and Postprandial Conditions in Hypertensive Patients: A Randomised Study

**DOI:** 10.3390/nu7021037

**Published:** 2015-02-04

**Authors:** Davide Grassi, Richard Draijer, Giovambattista Desideri, Theo Mulder, Claudio Ferri

**Affiliations:** 1 Department of Life, Health and Environmental Sciences, University of L’Aquila, Viale San Salvatore, L’Aquila 67100, Italy; E-Mails: giovambattista.desideri@cc.univaq.it (G.D.); claudio.ferri@cc.univaq.it (C.F.); 2 Unilever R & D, Olivier van Noortlaan 120, AT, Vlaardingen 3133, The Netherlands; E-Mails: Richard.Draijer@unilever.com (R.D.); Theo.Mulder@unilever.com (T.M.)

**Keywords:** black tea, flavonoids, hypertension, arterial stiffnesss, cardiovascular protection

## Abstract

Hypertension and arterial stiffening are independent predictors of cardiovascular mortality. Flavonoids may exert some vascular protection. We investigated the effects of black tea on blood pressure (BP) and wave reflections before and after fat load in hypertensives. According to a randomized, double-blind, controlled, cross-over design, 19 patients were assigned to consume black tea (129 mg flavonoids) or placebo twice a day for eight days (13 day wash-out period). Digital volume pulse and BP were measured before and 1, 2, 3 and 4 h after tea consumption. Measurements were performed in a fasted state and after a fat load. Compared to placebo, reflection index and stiffness index decreased after tea consumption (*p* < 0.0001). Fat challenge increased wave reflection, which was counteracted by tea consumption (*p* < 0.0001). Black tea decreased systolic and diastolic BP (−3.2 mmHg, *p* < 0.005 and −2.6 mmHg, *p* < 0.0001; respectively) and prevented BP increase after a fat load (*p* < 0.0001). Black tea consumption lowers wave reflections and BP in the fasting state, and during the challenging haemodynamic conditions after a fat load in hypertensives. Considering lipemia-induced impairment of arterial function may occur frequently during the day, our findings suggest regular consumption of black tea may be relevant for cardiovascular protection.

## 1. Introduction

Hypertension is the leading risk factor for cardiovascular morbidity and mortality [1]. Increased peripheral resistance due primarily to changes in vascular structure and function appear to be the fundamental hemodynamic abnormality in hypertension [2]. These changes include endothelial dysfunction, arterial wall thickening and abnormal vascular tone, and are due to alterations in the biology of the arterial wall [2]. Accordingly, an increased arterial stiffness is related to haemodynamic modifications at the level of the aorta, leading to a rise in cardiac afterload, a reduction in coronary perfusion and an over-stretch of the aortic wall [3,4]. The principal cause of increased systolic and pulse blood pressure (BP) is increased stiffness of the elastic arteries as a result of degeneration and hyperplasia of the arterial wall [3,4]. As stiffness increases, the reflected pulse wave amplitude increases and augments pressure in late systole, producing an increase in left ventricular afterload and myocardial oxygen demand. Current evidence reports that arterial stiffness should be recognized as a bidirectional interplay between the central and peripheral arteries. Specifically, the pressure pulse wave is not only transmitted forward to the periphery but also reflected backward to the central aorta. Consistent with this, also the pulse wave is composed of the forward and reverse components. Aortic stiffening and arteriolar remodeling due to hypertension not only augment the central pressure by increasing the wave reflection but may also alter the central bidirectional flow, inducing hemodynamic damage/dysfunction in target organs. Therefore, central hemodynamic monitoring has the potential to provide a diagnostic and therapeutic basis for preventing systemic target organ damage and for offering personalized therapy suitable for the arterial properties in each patient with hypertension [3,4,5]. An increasing number of studies have indicated arterial stiffness and the amount of pulse wave reflections as independent predictors of cardiovascular events and cardiovascular mortality in patients with different co-morbidities and cardiovascular risk [3,4,5]. Epidemiological studies have shown an inverse association between diets rich in flavonoids and cardiovascular disease [6] as well as on specific flavonoid intake and vascular function [7]. In this context, tea products account for a significant proportion of total flavonoid intake in different Western countries [8,9,10]. Hodgson *et al.* reported that regular ingestion of black tea over 6 months results in lower 24-h SBP and DBP [11]. Accordingly, we performed a dose-finding study showing that black tea intake decreased office systolic (−2.6 mmHg) and diastolic (−2.2 mmHg) BP as well as peripheral arterial stiffness [12].

However, a meal rich in fat has been reported to negatively affect BP and vascular function [13,14]. As most of the day is spent in the postprandial state, it is of interest to determine whether a fat-rich meal affects the postprandial BP and arterial haemodynamics. Further, the effect of treatment with black tea on vascular responses and on lipemia-induced impairment of arterial function has not been examined. Therefore, we aimed to investigate the effects of regular consumption of black tea, naturally rich in flavonoids, on wave reflections, systolic BP (SBP) and diastolic BP (DBP) before and after an oral fat load in untreated grade 1 hypertensive patients without additional cardiovascular risk factors.

## 2. Methods

### 2.1. Subjects

Nineteen untreated essential hypertensive (EH) patients (5 males and 14 females; mean age 51.5 ± 8.4 years) referred to our outpatient unit Centre of Hypertension and Cardiovascular Prevention of L’Aquila were recruited. Entry criteria were:≥ 18 and ≤ 75 years of age; no diabetes; SBP between 140 and 159 mmHg or DBP between 90 and 99 mmHg; body mass index between 19 and 31 kg/m^2^. Individuals were excluded if they had an acute or chronic disease, including any type of metabolic abnormality and/or major cardiovascular risk factors. Habitual smokers and use of any prescribed medication and/or dietary supplement within 2 weeks of entering the study were excluded. Female participants not in post-menopausal phase were also excluded. To further limit potential confounding factors, individuals were excluded if they reported daily intense sports activities (10 hours/week), changes of 10% body weight within 6 months of entering the study, current dietary treatment regimen, and/or participation in another clinical study within 3 months of entering this trial. The local Ethics Committee of L’Aquila approved the study in 20/12/2007 (ref: 53/2007), all clinical investigation have been conducted according to the principles expressed in the Declaration of Helsinki and all participants gave written informed consent.

### 2.2. Diagnosis of EH

Grade I EH was diagnosed according to European Societies of Hypertension and Cardiology criteria [15]. For this purpose, before enrolment into the study, BP and heart rate were measured after 10 min in a seated position in a comfortable room. SBP/DBP for inclusion in the protocol were 140/90 to 160/100 mmHg on 4 visits performed at 1-week intervals. During each visit, BP was measured in quadruplicate with an oscillometric device (Omron 705 CP, Omron) at 2-min intervals. The first BP reading was discarded and the average of the last three measurements was calculated. On each occasion, BP was recorded by the same physician who was unaware of the study design, objectives, and results (*i.e.*, was not a member of the research team). Secondary hypertension was excluded by clinical examination and appropriate tests.

### 2.3. Study Design

After a 7-days run-in period, participants were randomly assigned to consume a hot beverage containing 150 mg tea polyphenols (equivalent to 129 mg flavonoids) [16] or placebo twice a day for eight days in a double blind cross-over design. Wash-out between the two treatments was 13 days. During these periods subjects were asked to maintain their normal diet and lifestyle avoiding consumption of tea, red wine, chocolate based products, dietary supplements or aspirin. Vascular function was assessed prior to and after consumption of the test beverage in a fasted state on day 7 and after an oral fat load on day 8 of each of the two intervention phases. To hide the identity of the subjects, all eligible patients received a personal code which was used throughout the study. Personal codes were randomized to one of the two treatment sequences. Pre-weighted portions of the test products were supplied to the patients in sachets made from laminated aluminium foil. Sachets were coded with a 4-digit number. Several different numbers were used both for placebo and active treatment to prevent that the code could be easily broken. Compliance was checked by a questionnaire. Volunteers consumed two doses per day: approximately one hour before lunch and one hour before dinner. They were carefully instructed to add the content of a sachet to 100–200 mL boiling hot water and stir the solution until the powder was completely dissolved. Addition of sugar, milk, lemon, *etc*. was not allowed. The product was consumed while it was still hot.

On test days the volunteers consumed the test product at the facility at the beginning of the test sequence (*t* = 0). The composition of the dried tea extract and placebo products is given in Table 1. Before the start of the study and after the first visit to the test facility patients received the intervention products (20 sachets: 15 to be consumed plus five spare sachets). The intervention products were ambient stable, and the participants were advised to store the products in a locked cupboard. Volunteers returned the excess sachets and the empty sachets before the test sequence on day 8. These returned materials were used as a proxy to check compliance.

**Table 1:** Composition of the test products (mg per dose).

	Placebo	Active Treatment
Polyphenols	0	150
Total Caffeine	37.3	37.3
Tea solids	-	497.5
Caffeine added	37.3	-
Caramel colour	90	-
Tea flavour	10	-
Sucrose	1362.7	1402.5
Total weight of sachet	1500	1900

On the morning of day 7 and day 8 of the two test periods, volunteers came to the facility early in the morning in a fasted state. On both days, baseline BP and Digital Volume Pulse (DVP) measurements were performed. Volunteers were subsequently asked to consume the test product. On day 7, all measurements were performed in a fasted state, while on day 8 volunteers also consumed ultra-heat-treated whipping cream (1 gram fat per kg bodyweight; for 100 mL of product: fats 27 g, carbohydrates 12.6 g, proteins 2.1 g; energy value 302 kcal) approximately 30 min after consuming the test product. The range of energy intakes was between 649 and 951 kcal. On day 7, DVP and BP measurements were performed before (*t* = 0) and 1, 2, 3 and 4 h after consumption of the test product in a supine position in a quiet, temperature-controlled (22°C–24°C) room by trained, certified staff, who were blind about the study protocol. This procedure was repeated on day 8, but now including a fat load consumed approximately 30 min after consumption of the test products. The study (participant recruitment and follow-up) was conducted between March 1, 2008 and March 1, 2011. The study was stopped from April 2009 to May 2010 due to the earthquake that occurred in L’Aquila.

All ongoing and related trials for this drug/intervention are registered.

### 2.4. Digital Volume Pulse Measurements

The reflection index (RI; height of the second peak divided by the height of the first peak in the digital volume pulse [DVP]), stiffness index (SI; subject height divided by the time delay between direct and reflected waves in the DVP), and the peak-to-peak time (PPT; time from pressure systolic pressure inflection point (if present) or otherwise first pressure wave peak to second pressure wave peak or inflection point) were evaluated by a validated finger photoplethysmographic device (Micro Medical, Gillingham, Kent, UK) [17]. Three consecutive recordings were obtained in the index finger of the non-dominant hand with participants resting 15 min in supine position. Measurements were repeated if the waveforms did not pass the automatic quality controls by the software.

### 2.5. Blood Pressure Measurements

Office BP measurements were recorded by a validated oscillometric device with appropriately sized cuffs (Omron 705 CP; Omron Matsusaka Co. Ltd., Matsusaka-City, Japan) on the dominant upper arm. For this purpose, patients rested 15 min in supine position. Then, the first BP measurement was discarded and the subsequent three consecutive BP readings, taken at 5 min intervals, were recorded. The average of these latter measures was considered for statistical analysis.

### 2.6. Statistical Evaluation

#### 2.6.1. Size of The Study Population

Power was based on results obtained in a previous dose-dependent cross-over study with 20 healthy subjects. In that study, mean systolic BP decreased 2.6 mmHg with a 3.2 standard deviation of the difference between two values for the same patient after the intake of tea with 400 mg of flavonoids per day. On the basis of these data, a similar size difference should be detected in a group of 18 individuals (two-sided, alpha = 0.05 and power = 0.9). In order to account for a 10% drop-out, we decided to include 20 volunteers. A randomization scheme was prepared by a statistician and the generation of the random allocation was done by computer generated random numbers. Randomisation was done before the start of the study by assigning treatment orders to subject numbers. It was a blocked randomization with a block size of 2 and an allocation ratio of 1:1. Sachets with the test products were labelled with subject number and treatment period. Treatments were supplied in sealed in envelopes labelled with the subject number. These were opened in consecutive order by each participant. Participants, statisticians, care providers and those assessing the outcome of the study were all blinded. Black tea and placebo were very similar in taste and appearance but were not identical. Subjects might have perceived that the products were different but should not have been able to deduce which product was the active treatment.

Data analysis was performed using the SAS software (SAS Institute, Cary North Carolina, version 9.1). Descriptive analysis consisting of distribution statistics (number of available observations, mean and standard deviations) were presented for continuous data. Differences between the experimental groups and the placebo of the acute effect after one week intervention were evaluated by means of an analysis of covariance, integrating the data of 1, 2, 3 and 4 hours after test product ingestion. The statistical model included the covariables baseline (*t*0), gender, age and BMI. Fixed factors included in the model: treatment, period, day, time and all their interactions. Other interactions included in the model: baseline–treatment, baseline–period, baseline–time. The chronic effects, after one week intervention, were calculated from the *t*0 data using a similar model, excluding the baseline as a covariable.

The influence of the interventions was measured within each individual. Therefore, the variation due to differences between individuals was separated from the relevant error variance in the analysis. A Dunnett test was performed in order to correct for multiple testing between the active treatment and the placebo with and without a fat load. The statistical analysis results are presented as LSmeans. Statistical tests are two-sided for all analyses with a significant level of 0.05.

## 3. Results

Baseline characteristics of the study participants are provided in Table 2. According to the inclusion/exclusion criteria, participants presented without additional cardiovascular risk factors.

Of the 20 individuals enrolled, 19 completed the study. One individual dropped out during the first study phase due to personal circumstances and was excluded from the statistical analysis. Compliance was 100% in all the volunteers for all the study phases.

**Table 2:** General characteristics of the study population (mean ± SD).

Characteristic	Value
Number of subjects (total/males)	19 / 5M
Age (years)	51.3 ± 8.2
BMI (Kg/m^2^)	27.1 ± 1.2
Body Weight (Kg)	73.7 ± 7.2
LDL-cholesterol (mg/dL)	141.1 ± 27.3
HDL-cholesterol (mg/dL)	45.7 ± 8.2
Triglycerides (mg/dL)	116.8 ± 38.1
Plasma glucose (mg/dL)	86.8 ± 7.3
Plasma insulin (μU/mL)	11.3 ± 5.4

### 3.1. Wave Reflections Analysis

**Figure 1:**
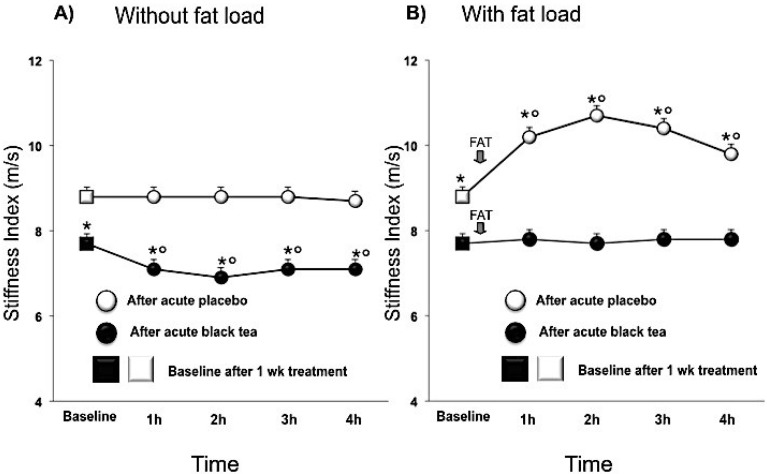
Effect of 1 week placebo (white) and black tea (black) administration on baseline (square) values (Panel **A** and **B**) of stiffness index and acute effects without and with fat load (Panel **A** and **B**). Data are presented as LSmeans ± SE. In all panels vertical lines indicate SE and asterisks (*) indicate significant differences with respect the placebo phase (Panel **A** and **B**) while circles (°) indicate significant differences from baseline values (Panel **A** and **B**). Differences are considered significant when *p* < 0.05.

**Figure 2:**
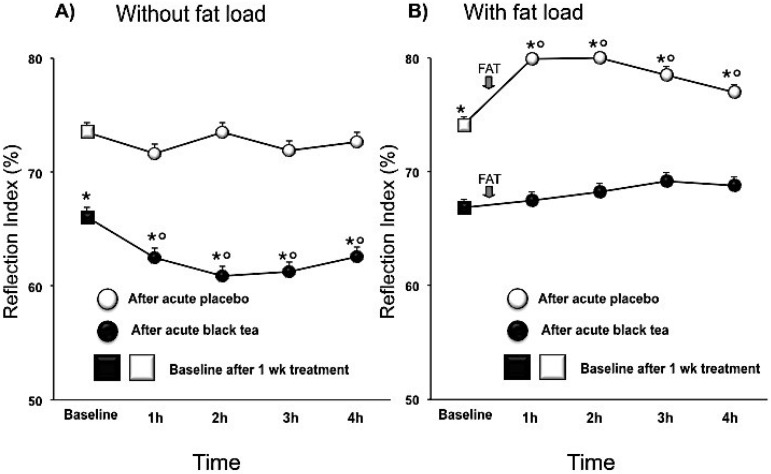
Effect of 1 week placebo (white) and black tea (black) administration on baseline (square) values (Panel **A** and **B**) of reflection index and acute effects without and with fat load (Panel **A** and **B**). Data are presented as LSmeans ± SE. In all panels vertical lines indicate SE and asterisks (*) indicate significant differences with respect the placebo phase (Panel **A** and **B**) while circles (°) indicate significant differences from baseline values (Panel **A** and **B**). Differences are considered significant when *p* < 0.05.

Compared to placebo, black tea ingestion affected pressure wave reflections after chronic intervention, lowering the SI (mean difference, 95% CI) by −1.2 (−1.8, −0.5; *p* < 0.001) m/s and the RI by −7.5 (−12.3, −2.6; *p* < 0.005) % and increasing PPT by 28.9 (13, 44.7; *p* < 0.001) ms. Over and above the chronic effect there was an acute effect of tea (Table 3; Figure 1A and Figure 2A). After fat load, RI increased immediately with significant effects being noted within 30 min (Figure 2B). SI also increased after the fat load, but peak effects were delayed to 90 min (Figure 1B). While in the fasted state, SI, RI and PPT improved with tea, and the negative effects of the fat challenge on these vascular parameters were negated by prior tea consumption (Figure 1B and Figure 2B).

### 3.2. Blood Pressure

**Figure 3:**
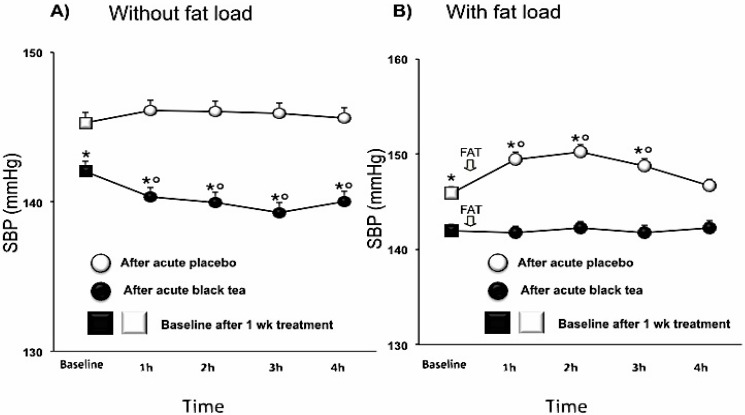
Effect of 1 week placebo (white) and black tea (black) administration on baseline (square) values (Panel **A** and **B**) of systolic blood pressure and acute effects without and with fat load (Panel **A** and **B**). Data are presented as LSmeans ± SE. In all panels vertical lines indicate SE and asterisks (*) indicate significant differences with respect the placebo phase (Panel **A** and **B**) while circles (°) indicate significant differences from baseline values (Panel **A** and **B**). Differences are considered significant when *p* < 0.05.

**Figure 4:**
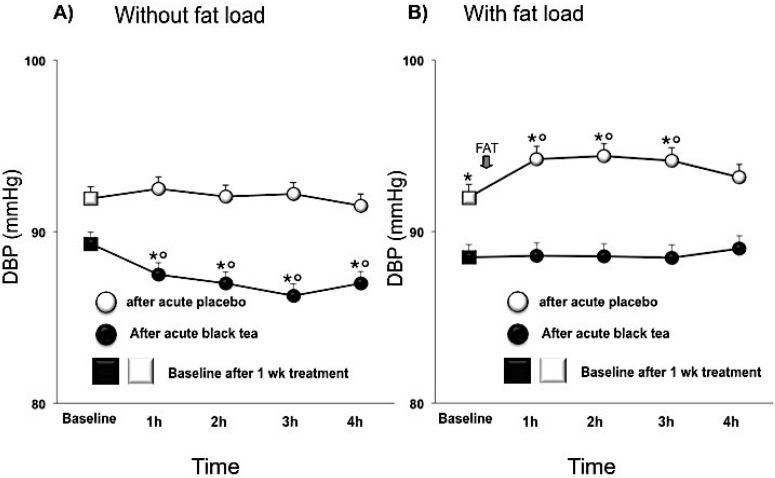
Effect of 1 week placebo (white) and black tea (black) administration on baseline (square) values (Panel **A** and **B**) of diastolic blood pressure and acute effects without and with fat load (Panel **A** and **B**). Data are presented as LSmeans ± SE. In all panels vertical lines indicate SE and asterisks (*) indicate significant differences with respect the placebo phase (Panel **A** and **B**) while circles (°) indicate significant differences from baseline values (Panel **A** and **B**). Differences are considered significant when *p* < 0.05.

After one week intervention, SBP was significantly decreased after black tea ingestion compared to placebo by −3.2 (−5.2,−1.2; *p* < 0.005) mmHg and DBP tended to be lower by −2.6 (−5.4, 0.2; *p* < 0.0001) mmHg. HR was not affected. The BP lowering effect was enhanced by an additional acute effect of tea (Table 3; Figure 3A and Figure 4A). The fat load increased BP, reaching its maximal effect about 90 min after intake, whereas, after black tea ingestion, SBP and DBP did not significantly rise after the fat load (Table 4; Figure 3B and Figure 4B).

**Table 3:** Effect of black tea on haemodynamic parameters after one week intervention measured in the fasted state.

parameter	placebo	black tea	difference (95% CI)	Adj *P*-value
Systolic BP (mmHg)	144.5 ± 0.2	141.1 ± 0.2	−3.3 (−4.3, −2.3)	<0.0001
Diastolic BP (mmHg)	90.6 ± 0.2	88.1 ± 0.2	−2.5 (−3.3, −1.8)	<0.0001
Heart rate (bpm)	72.0 ± 0.3	70.8 ± 0.3	−1.2 (−2.3, 0.0)	<0.05
Pulse Pressure (mmHg)	53.9 ± 0.2	53.6 ± 0.2	−0.3 (−1.3, 0.7)	n.s.
Peak to peak time (ms)	198.9 ± 2.2	224.1 ± 2.3	25.1 (15.6, 34.6)	<0.0001
Reflection Index (%)	69.9 ± 0.7	64.6 ± 0.6	−5.2 (−7.9, −2.6)	<0.001
Stiffness Index (m/s)	8.2 ± 0.1	7.5 ± 0.1	−0.7 (−1.0, −0.5)	<0.0001

Values are LSmeans ± SEM, difference tea-placebo with 95% confidence interval (95% CI); Data are the mean values calculated from *t*1, 2, 3 and 4 h after the last test dose, and analyzed by ANCOVA with the baseline value, gender, age and BMI taken as covariables; BP = blood pressure; n.s. = not significant.

## 4. Discussion

The novel finding of this study is that consumption of black tea, naturally rich in flavonoids, compared to a surrogate placebo resulted in significantly lower wave reflections and BP. Furthermore, the black tea counteracted or completely prevented the abnormalities in peripheral arterial haemodynamics and BP which were caused by a fat load in grade I hypertensive patients. These effects were observed with a tea dose of 2 cups per day, thus easily reachable in normal daily life.

**Figure 5:**
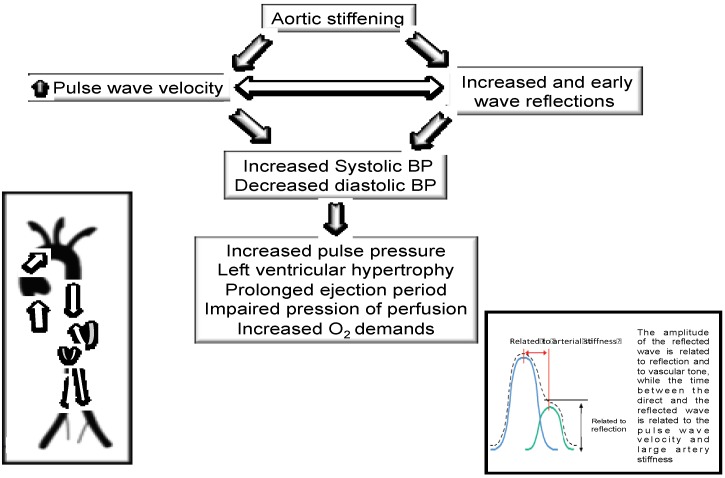
Arterial stiffness and wave reflections are known to play a fundamental role in cardiovascular health and disease. Indeed, in interplay with left ventricular ejection and the elastic properties of the aorta, wave reflections could specifically increase central pulse pressure, a recognized predictor of cardiovascular risk. These changes are attributed to the timing and amplitude of pulse wave reflections from peripheral reflecting sites, where high resistance arterioles are considered to be the major sites of wave reflection in the circulation.

The structural and functional changes in the arterial roundtrip travel time of the pressure and flow wave from the heart to the periphery and back is directly related to major reflecting site distance and inversely related to the pulse wave velocity [2,3]. Increased arterial stiffness causes an increase in transmission velocity of both forward and reflected waves, which, in turn, causes the reflected wave to arrive earlier in the central aorta with greater amplitude and duration. These changes in wave reflection characteristics augment pressure and decrease flow in late systole. These modifications in arterial effects and reflected wave characteristics cause an increase in systolic (and pulse) BP and left ventricular force generation, which cause a reduction in stroke output [2,3]. Similar changes occur in the shape of pressure and flow waves when the elastic arteries stiffen. The reflected wave arrives at the heart early during systole because of increased pulse wave velocity and decreased travel time of the reflected wave from the periphery (Figure 5). The structural and functional changes in the arterial circulation provide disturbances in regional blood flow, progression of atherogenesis, and microvascular changes occurring during senescence and in the presence of cardiovascular risk factors [2,3,18] (Figure 5).

**Table 4:** Effect of black tea on haemodynamic parameters after 8 days’ intervention measured in the postprandial state.

parameter	placebo	black tea	difference (95% CI)	Adj *P*-value
SBP (mmHg)	146.7 ± 0.2	143.5 ± 0.2	−3.2 (−4.2, −2.2)	<0.0001
DBP (mmHg)	92.5 ± 0.2	90.5 ± 0.2	−2.0 (−2.7,−1.2)	<0.0001
HR (bpm)	71.4 ± 0.3	71.4 ± 0.3	−0.0 (−1.2, 1.1)	n.s.
PP (mmHg)	54.3 ± 0.2	53.4 ± 0.2	−1.0 (−2.0, 0.0)	n.s.
PPT (ms)	170 ± 2.3	203 ± 2.2	33.9 (24.6, 43.3)	<0.0001
RI (%)	76.3 ± 0.7	70.4 ± 0.6	−5.9 (−8.5, −3.3)	<0.0001
SI (m/s)	9.8 ± 0.1	8.3 ± 0.1	−1.5 (−1.8, −1.2)	<0.0001

Values are LSmeans ± SEM, difference tea-placebo with 95% confidence interval (95% CI); Data are the mean values calculated from *t*1, 2, 3 and 4 h after the last test dose, and analyzed by ANCOVA with the baseline value, gender, age and BMI taken as covariables; SBP = systolic blood pressure, DBP = diastolic blood pressure, HR = Heart rate, PP = pulse pressure, PPT = peak to peak time, RI = reflection Index, SI = stiffness index. n.s. = not significant.

Changes in wave reflection properties are associated with vascular disease and aging and cause an increase in left ventricular afterload, myocardial mass, and oxygen consumption [3]. Hypertensive disease is associated with important structural alterations in the vasculature, such as large artery stiffening, small artery remodeling and microvascular rarefaction [2,3]. Recent basic research has revealed some of the molecular pathways involved in the remodelling of the cardiovascular system are under the influence of physical forces. Vasoactive drugs have little direct effect on large elastic arteries, but can markedly change wave reflection amplitude and augmentation index by altering stiffness of the muscular arteries and modifying transmission velocity of the reflected wave from the periphery to the heart [2,3,4,5]. Furthermore, arterial stiffening is the principal cause of increasing SBP with advancing years and in patients with arterial hypertension [2,3,4,5]. It is correlated with progressive arterial dilation as a consequence of arterial wall degeneration, probably due to repetitive cyclic mechanical stress [2,3,4,5]. The increased amplitude of the pressure wave generated by a given flow impulse from the heart, and indirectly by increasing wave velocity so that wave reflection from the periphery occurs earlier, and augments pressure in late systole. The first mechanism affects pressure in both the central and peripheral arteries, the second predominantly in the central arteries [2,3,4,5]. This study used DVP wave analysis to measure changes in vessel tone and wave reflections. The RI is an index of pressure wave reflection and vascular tone and SI of wave reflections and arterial stiffness [17,18]. Moreover, the marked changes that appear in RI in response to vasoconstrictors and vasodilators has been considered moderately correlated with pulse wave velocity (PWV) (*r* = 0.65) [18]. Although SI is a measure of wave reflections that increases with age, it has also been described to be sensitive to small changes in vascular tone induced by vasodilators [17,18]. Furthermore, although SI was found to be inappropriate as a surrogate of aortic PWV [19], assessment of the SI derived by digital photoplethysmography has been considered an advantage in risk stratification of subjects with intermediate and high cardiovascular risk compared with the AIx [20].

In this study, we observed that black tea ingestion decreased BP, SI and RI, and protected arterial haemodynamic alteration induced by a fat load. Overall, we showed decreased peripheral vascular tone and wave reflections, suggesting that tea consumption caused vasodilation of peripheral arteries indicating a decrease of vascular resistance, thus offering a putative mechanism for the reported effects in reducing BP. According to this, we may suppose that flavonoid-rich black tea was able to positively affect arterial function also by improving endothelial function [12].

Loss of functional integrity of the vascular endothelium may be one of the initiating events in the etiology of atherosclerosis. Endothelial cells interact with blood components and the abluminal tissues, thus playing an active role in many aspects of vascular functions, such as permeability and vessel tone regulation. Endothelial cells are constantly exposed to nutrients which can modulate enzymes, receptors, transport molecules and various vasoactive mediators, resulting in significant functional changes of the endothelium and the underlying tissues [21,22]. There is evidence that individual nutrients or nutrient derivatives may either provoke or prevent metabolic and physiologic perturbations of the vascular endothelium [21,22,23,24]. Diets high in fat and/or calories are considered a risk factor for the development of atherosclerosis. Accordingly, certain diet-derived lipids and their derivatives can disrupt normal endothelial integrity, thus reducing the ability of the endothelium to act as a selectively permeable barrier to blood components [21,22,23,24]. Mechanisms underlying fatty acid-mediated endothelial cell dysfunction may be related to changes in fatty acid composition as well as to an increase in cellular oxidative stress [21,22,23,24]. Selective lipid accumulation and fatty acid changes in endothelial cells can modulate membrane fluidity, proteoglycan metabolism and signal transduction mechanisms. Most importantly, dietary fats rich in certain unsaturated fatty acids may be atherogenic by enhancing the formation of reactive oxygen intermediates. A subsequent imbalance in cellular oxidative stress/antioxidant status can activate oxidative stress-responsive transcription factors, which in turn may promote endothelial activation with expression of adhesion molecules on the surface of endothelial cells, and thus intensify an inflammatory response in atherosclerosis. Hypertriacylglycerolaemia in the postprandial state promotes the formation of small, dense low-density lipoproteins, as well as oxidative stress, inflammation, and endothelial dysfunction, all of which compound the risk of cardiovascular disease [21,22,23,24]. Because in normal subjects blood concentrations of glucose, lipids and insulin are increased after each meal, and postprandial changes last a long time after the meals, these changes might be of importance in the process of atherosclerosis initiation and development. These mechanisms may be involved in the development of atherosclerosis in normal subjects when food intake is chronically modified towards glucids and lipids with cumulative effects both on depression of endothelium dependent dilation and oxidative stress [21,22,23,24].

Impaired endothelium-dependent vasodilation due to decreased bioavailability of nitric oxide has been reported after a fat load in both healthy and obese individuals [14,23]. In particular, it has been observed that acute fat load administered orally or intravenously significantly increased BP, altered endothelial function, and activated the sympathetic nervous system by mechanisms not related to changes in leptin, glucose, and insulin levels [14,23]. Similarly, combined glucose and fat loads had additional deleterious impact on flow-mediated dilation and on nitric oxide bioavailability in hypertensive patients [24]. Thus, fat load, independent of its source, has deleterious hemodynamic effects characterized by increased cardiovascular risk in hypertensive as well as in normal subjects [14,23,24]. Our study was conducted in patients affected by EH and indicated that black tea is not only able to improve vascular function and BP, but also counteracts fat load induced transient vascular impairment resulting in a parallel increase in BP and wave reflection. From a public health point of view, avoiding to suggest black tea consumption as a ticket to consume high-fat diets, these findings may be of relevance: the tested tea dose of 1 (acute) or 2 cups (per day) was moderate and the intervention time relatively short. Moreover, the average energy intake in this study of 824 kcal mimicked a serving size typical for the main meal in Western diets [25,26]. The magnitude of wave reflection and arterial stiffness has been shown to have an independent predictive value for all cause mortality and cardiovascular morbidity, coronary events and strokes in hypertensive patients [27]. Also, small changes in BP—for instance, due to dietary and lifestyle modification­may have a significant impact on the prevalence of hypertension and risk of cardiovascular disease [28].

## 5. Conclusions

We observed for the first time that black tea, naturally rich in flavonoids, counteracted or completely prevented the abnormalities in peripheral arterial haemodynamics that were caused by an acute oral fat load in never-treated hypertensive patients. It is important to note that while our study design was rigorous, it was also short-term and included a small number of subjects. A review of epidemiological and mechanistic studies suggests that flavonoids from different sources manifest beneficial effects on the cardiovascular system. Several lines of clinical and experimental evidence also indicate that tea may help to reduce cardiovascular disease risk by improving endothelial function and decreasing BP levels. This may significantly contribute to the cardiovascular disease incidence, when one considers that tea is globally the most consumed beverage after water.
